# Impact of chitosan oligosaccharide on microbiota-metabolite-immune axis in natural aging

**DOI:** 10.3389/fnut.2025.1722269

**Published:** 2026-01-23

**Authors:** Wei Liu, Pengcheng Shi, Yunyu Xiao, Xi Yang, Baoming Tian

**Affiliations:** 1 Zhejiang Provincial Key Laboratory of Agricultural Microbiomics, Institute of Plant Protection and Microbiology, Zhejiang Academy of Agricultural Sciences, Hangzhou, China; 2 Zhejiang Golden Shell Pharmaceutical Co., Ltd, Yuhuan, China; 3 Fuyao University of Science and Technology, Fuzhou, Fujian, China; 4 College of Food Science and Technology, Zhejiang University of Technology, Huzhou, China

**Keywords:** chitooligosaccharide, gut microbiota, inflammation, Muribaculaceae, natural aging, propionate metabolism

## Abstract

Natural aging involves an imbalance in gut bacteria, changes in metabolism, and mild ongoing inflammation. The integrated impact of chitosan oligosaccharide (COS) on microbiota-metabolite-immune interactions in a physiological (non-accelerated) aging context remains unclear. In this study, male C57BL/6J mice (young control; aged vehicle; aged +COS from 10 to 18 months) underwent longitudinal assessment including 16S rRNA profiling, untargeted serum metabolomics, multiplex cytokine measurement, and colonic p53 and p21 immunohistochemistry. The results showed that COS was associated with restructuring of gut community composition, with reduced Firmicutes (67.07% to 32.93%) and increased *Bacteroidota* (15.29% to 31.22%), alongside marked enrichment of *Muribaculaceae* (to 52.83%). Discriminant metabolites (VIP > 1 and FDR-adjusted) mapped predominantly to propionate (propanoate) and amino acid-linked pathways. Integrative correlation analysis connected *Muribaculaceae* with propionate-associated and aromatic amino acid-related metabolites and selected cytokines (including CCL20). COS mitigated age-associated body weight gain and was accompanied by reduced p53 and p21 immunoreactivity in brain and kidney, consistent with attenuation of stress associated senescence signaling. Long-term COS supplementation in naturally aged mice is associated with coordinated shifts in a putative *Muribaculaceae*-propionate-immune axis and concurrent down-regulation of both p53 and p21. These associative findings warrant mechanistic validation through targeted short-chain fatty acid quantification, receptor signaling assays, microbiota transfer, and functional aging endpoints to clarify causality and translational potential.

## 1 Introduction

Global population aging is accelerating, imposing increasing pressure on health care systems and socioeconomic sustainability (1, 2). Biologically, aging reflects a gradual loss of the body's ability to maintain balance rather than a linear consequence of a single lesion. The hallmarks of aging framework, comprising genomic instability, telomere attrition, epigenetic changes, loss of protein homeostasis, disrupted nutrient sensing, mitochondrial dysfunction, cellular senescence, stem cell exhaustion, and altered intercellular communication, provides a comprehensive foundation for mechanistic investigation and therapeutic intervention (3). Among these dimensions, chronic low-grade inflammation amplifies cumulative molecular and cellular damage, escalating risk for degenerative and metabolic disease states (4, 5).

The gut microbiota undergoes compositional and functional remodeling with age, influencing barrier integrity, immune calibration, and metabolic signaling loops implicated in inflammation (6–9). Identifying interventions that concurrently modulate microbial ecology and host inflammatory or oxidative stress axes is therefore of translational interest. Chitosan oligosaccharide (COS), a low-molecular weight, water soluble derivative of chitin/chitosan, has attracted attention because of its favorable safety profile and multi-target properties (10). Experimental studies indicate that COS attenuates oxidative injury, inflammatory signaling, and tissue damage in D-galactose-induced accelerated paradigms (11); mitigates intestinal inflammation under lipopolysaccharide challenge partly via calcium-sensing receptor-related pathways (12); and modulates human gut microbial composition with ancillary antiglycation potential in *ex vivo* or controlled settings (13). Collectively, these observations suggest that COS may act along an integrated microbiota–metabolite–immune axis relevant to aging biology.

However, much of the existing evidence derives from chemically accelerated models. The D-galactose paradigm, while experimentally tractable and characterized by oxidative stress and advanced glycation end product accumulation, does not reproduce the temporal layering, cross-tissue signaling complexity, or ecological progression of natural aging (14–16). Thus, extrapolating COS-associated benefits from accelerated conditions to physiological aging remains provisional.

Mechanistic elucidation in a natural aging context requires cross-domain integration. High-resolution metabolomics can sensitively detect early metabolic drift in energy- and redox linked intermediates (16). Concurrently, recent mapping of indicative microbial functional taxa in naturally aging mice shifts interpretation beyond purely taxonomic inventories toward ecologically coherent consortia with emergent functional implications (17).

Despite foundational evidence for the anti-inflammatory and microbiota-modulating potential of COS, significant knowledge gaps remain:

(1) It is unclear whether, during natural (non-accelerated) aging, COS is associated with the restructuring or stabilization of specific functional bacterial groups, such as polysaccharide-degrading and short-chain fatty acid–linked consortia, which influence immune-metabolic tone (17).

(2) The relationship between microbiota-centered and short chain fatty acid-linked immune and barrier modulation pathway that may affect low-grade inflammatory status is not well understood (7, 17).

(3) It is uncertain whether ecological and metabolic changes extend to cellular energy and stress adaptation mechanisms, such as e.g., NAD+-related deacetylation dynamics and mitochondrial quality control, which are crucial for proteostasis and redox balance (16, 18).

(4) The degree to which COS-associated molecular signatures demonstrate translational coherence between accelerated and natural aging contexts remains to be determined (11, 15).

To address these questions, we implemented an integrated analytical pipeline combining (a) 16S rRNA gene–based microbiota profiling, (b) functional guild inference, (c) untargeted serum metabolomics with emphasis on inflammatory and redox-sensitive metabolites, and (d) inflammatory and oxidative stress phenotype assessment, situated within a natural aging murine model. By juxtaposing outcomes with prior accelerated model observations (14, 15), we aimed to refine mechanistic mapping of COS within a physiologically authentic aging milieu.

We hypothesized that long-term COS supplementation would be associated with (i) enrichment of SCFA-associated microbial communities (notably *Muribaculaceae*), (ii) remodeling of propionate (propanoate)- and amino acid-linked serum metabolite pathways, (iii) modulation of cytokine profiles, and (iv) attenuation of colonic p53 and p21 immunoreactivity in naturally aged mice. In delineating these multilayer associations, this study seeks to inform low-risk, ecology responsive strategies for mitigating inflammation and functional decline (17).

## 2 Materials and methods

### 2.1 Study design and ethics

Chitooligosaccharide (COS) was obtained from Zhejiang Golden-Shell Pharmaceutical Co., Ltd. (Yuhuan, Zhejiang, China). The COS (Batch No. M-KG-2504001) had an average molecular weight of < 1000 Da and a purity (content) of 95.1%. The substance was stored in a dry, light-resistant container and dissolved in distilled water immediately before administration.

**Figure 1:**
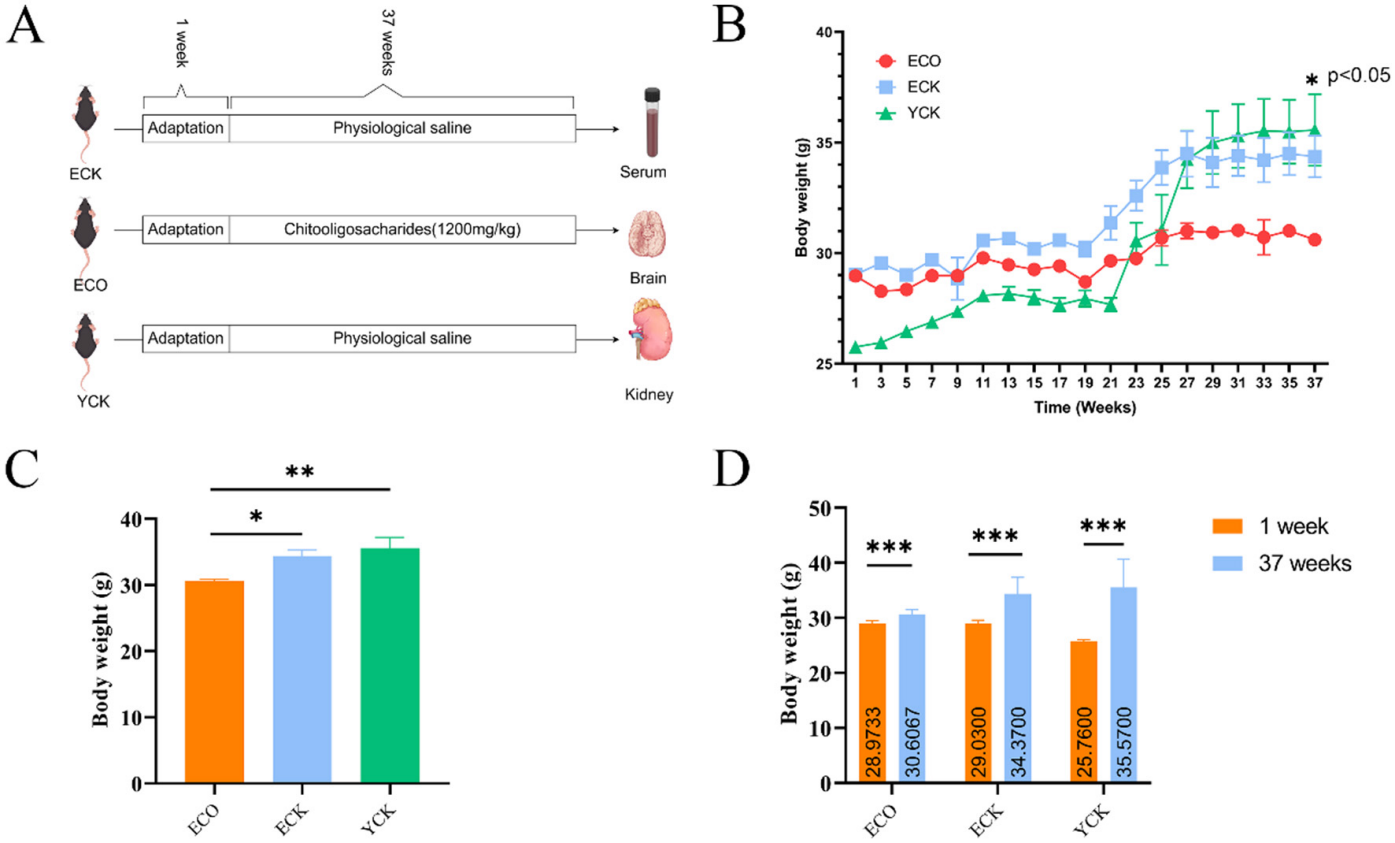
Study design and effects of chitosan oligosaccharide (COS) on body weight in naturally aged mice. **(A)** Experimental timeline and group allocation. Male C57BL/6J mice entered the study at 10 months (aged cohorts) or 3 months (young cohort). Aged mice received vehicle (ECK) or COS (ECO; 1,200 mg/kg/day) for the intervention period; young controls (YCK) received vehicle. **(B)** Longitudinal body weight trajectories (mean ± SEM), showing attenuation of age associated weight gain in ECO. **(C)** Net body weight gain (terminal minus baseline). **(D)** Baseline vs. terminal body weight within each group. Statistical analysis by one-way ANOVA **(C, D)**; symbols indicate significant differences (e.g., ^*^p < 0.05 vs. ECK; ^**^p < 0.01, ^***^p < 0.001). YCK, young control; ECK, aged control; ECO, aged + COS; COS, chitosan oligosaccharide.

A long-term intervention study was conducted to evaluate the effects of chitosan oligosaccharide (COS) on aging-related parameters in male C57BL/6 mice. COS (degree of polymerization:2-10) was obtained from Zhejiang Gold Shell Pharmaceutical Co. Ltd. (Yuhuan, China). The intervention lasted 8 months (Figure 1) with continuous standard diet feeding. Ethical approval was granted by the Ethics Committee of the Zhejiang Academy of Agricultural Sciences (Approval No. 2021ZAASLA72). All procedures com-plied with national regulations for laboratory animal welfare. Mice were euthanized by cervical dislocation.

### 2.2 Animals, housing, and diet

Male C57BL/6 mice aged 10 months (28–30 g) and a young cohort aged 3 months (25–26 g) were acquired from Hangzhou Ziyuan Laboratory Animal Technology Co., Ltd, Hangzhou, China. After a 7-day acclimatization period, all miceanimals were housed in cages under controlled environmental conditions (temperature: 25 ± 2 °C, relative humidity 50 ± 5%, 12-h light/dark cycle) and provided, with *ad libitum* access to standard rodent breeding diet (XT108FZ-001, Xietong Biotechnology, Nanjing, China) and autoclaved water throughout the experiment. The breeding diet was selected as the facility's standard chow to support reproduction, growth, and maintenance (macronutrient composition: approximately 24% crude protein, 12.9% fat, 63% carbohydrates; metabolizable energy 3.5 kcal/g).

### 2.3 Experimental groups and randomization

Mice were allocated to three groups: young control (YCK, *n* = 10), aged control (ECK, *n* = 10), and aged COS (ECO, *n* = 15). Randomization was performed after acclimatization for 7 days using a computer-generated random number sequence in R with simple block randomization (block size = 5) stratified by baseline body weight (±1 g) to balance initial body weight across groups. Cage allocation avoided housing all animals from one group in a single cage to reduce cage (microenvironment) confounding. Investigators performing downstream analyses of histology and image quantification were blinded to group identity. While the full cohort was utilized for general physiological monitoring (e.g., body weight), a representative sub-cohort was randomly selected for downstream molecular and omics analyses due to sample availability and experimental constraints. The specific sample size (*n*) for each analysis is indicated in the corresponding figure legends.

### 2.4 Monitoring, sample collection, and processing

Body weight was recorded twice weekly. Blood collection (retro-orbital) occurred at designated time points under mild isoflurane anesthesia. After clotting (2 h, room temperature; then 2–3 h at 4 °C), samples were centrifuged (3,000 g, 10 min), and serum stored at −80 °C. Following sacrifice cecal contents were collected in sterile tubes and frozen (−80 °C) until microbiome and short-chain fatty acid (SCFA) analysis. No antibiotics were administered during the intervention period.

### 2.5 Immunohistochemistry for p53 and p21

Paraffin-embedded colonic sections (4 μm) were deparaffinized, rehydrated, and subjected to high-pressure antigen retrieval (3 min) in 10 mM sodium citrate buffer (0.05% Tween-20, pH 6.0). Endogenous peroxidase was quenched with 3% H2O2 (20 min, room temperature). After phosphate-buffered saline washes, sections were incubated with primary antibodies against p53 and p21 (each 1:100; p53, Cell Signaling Technology, cat. #2524; p21, Cell Signaling Technology, cat. #2947) overnight at 4 °C in a humidified chamber. Detection used an HRP-conjugated secondary system (EnVision+ System-HRP, DAB; Agilent/Dako, cat. K4007) with DAB chromogenic development; hematoxylin counterstaining followed. Slides were dehydrated, cleared, and mounted. Semi-quantitative evaluation employed the H-score method (18): *H*-score = Σ (Pi × Ii), where Pi is the per-centage (0–100%) of cells at a given staining intensity (Ii = 0, 1, 2, 3). At least 6 non-overlapping high-power fields (HPFs, 400 × ) per sample were analyzed. Two independent observers blinded to group identity performed scoring; discrepancies >10% were re-reviewed to consensus. Adjacent negative controls (omission of primary antibody) were included in each staining batch.

### 2.6 Cytokine profiling

Serum cytokines were quantified using the RayBio Quantibody Mouse Th17 Array 1 (RayBio, Norcross, GA, USA) per manufacturer's instructions. The panel included IL-1β, IL-2, IL-4, IL-5, IL-6, IL-10, IL-12p70, IL-13, IL-17A, IL-17F, IL-21, IL-22, IL-23, IL-28, IFN-γ, MIP-3α (CCL20), TGF-β1, and TNF-α. Arrays were scanned (GenePix 4400) and processed with GenePix Pro. Concentrations were interpolated from standard curves (five or more calibration points). Technical duplicates were averaged; coefficients of variation (CV) >15% triggered re-examination.

### 2.7 16S rRNA gene sequencing and bioinformatic processing

Genomic DNA was extracted from cecal contents using the FastDNA Spin Kit for Soil (MP Biomedicals, Santa Ana, CA, USA) following the protocol for high-complexity microbial samples. The V3–V4 region was amplified with primers 338F and 806R. PCR products were purified (PCR Clean-Up Kit, Yuhuan, Shanghai, China), quantified (Qubit 4.0; Thermo Fisher Scientific, Waltham, MA, USA), and pooled equimolarly. Sequencing was performed on an Illumina NextSeq 2000 platform (Illumina, San Diego, CA, USA) (2 × 250 bp) at Shanghai Majorbio Bio-Pharm Technology Co., Ltd. Bioinformatic processing was executed via the Majorbio Cloud Platform (https://www.majorbio.com). Quality filtering removed reads with ambiguous bases or average quality < Q20. Paired reads were merged, and chimeras filtered. Features were resolved as amplicon sequence variants (ASVs) via DADA2. Taxonomy was assigned against the Silva database (version 138) with a minimum confidence threshold of 0.7. Samples were rarefied to 30,000 reads to standardize alpha diversity comparisons. Alpha diversity (Sobs, Chao, Shannon, Goods coverage) was calculated with Mothur v1.30.1 (19). Beta diversity was assessed using Bray–Curtis distance matrices with Principal Coordinate Analysis (PCoA). Group separation was tested by PERMANOVA (999 permutations). Differential taxa were identified by LEfSe (LDA > 2, *p* < 0.05) ADDIN EN.CITE (20). Sequencing data are deposited in the NCBI SRA under accession PRJNA1328410.

### 2.8 Cecal content short-chain fatty acid quantification

SCFAs (acetate, propionate, butyrate, isobutyrate, valerate, isovalerate) were quantified by gas chromatography using a DB-FFAP column (0.32 mm × 30 m × 0.5 μm). Approximately 50 mg cecal samples were homogenized and diluted 10-fold in 0.1 mM PBS (phosphate-buffered saline, pH 7.0) containing crotonic acid as internal standard. After acidification with metaphosphoric acid–crotonic acid, samples were incubated at −30 °C for 24 h, centrifuged, filtered (0.22 μm), and injected (1 μL; split ratio 8:1). Injector and detector temperatures were 250 °C. The oven temperature program ramped from 80 °C to 230 °C. Flame ionization detector conditions: nitrogen 30 mL/min, hydrogen 40 mL/min, air 400 mL/min. SCFA concentrations were calculated from external calibration curves (*R*^2^ > 0.99).

### 2.9 Serum untargeted metabolomics

Untargeted metabolomics was performed on serum (ECK *n* = 5; ECO *n* = 5) by Shanghai Biotree Biotech Co., Ltd (Shanghai, China). Samples were processed with internal standards and normalized to sample mass. A pooled quality control (QC) sample was injected every 10 analytical runs. Chromatographic separation employed a Vanquish UHPLC (Thermo Fisher Scientific) with a Waters ACQUITY UPLC BEH Amide column (2.1 mm × 50 mm, 1.7 μm). Detection used an Orbitrap Exploris 120 mass spectrometer (positive and negative electrospray modes). Key source parameters: sheath gas 50 Arb, auxiliary gas 15 Arb, capillary temperature 320 °C, spray voltage +3.8 kV/−3.4 kV. Full MS resolution was 60,000; MS/MS resolution 15,000 with stepped normalized collision energies (20/30/40). Raw data were processed for peak picking, alignment, and deconvolution using Compound Discoverer with QC-based drift correction. Metabolite annotation lever-aged exact mass, retention time, and MS/MS spectral matching against in-house and public databases (KEGG, HMDB); identifications followed the Metabolomics Standards Initiative (MSI) with Level 2 confidence unless otherwise noted. Pathway enrichment utilized KEGG and MetaboAnalyst. Differential metabolites were defined by OPLS-DA variable importance in projection (VIP) > 1 combined with Benjamini–Hochberg false discovery rate (FDR)-adjusted *p* < 0.05 (Student's *t*-test or Welch's *t*-test based on variance).

### 2.10 Statistical analysis, quality control, and data availability

Data are presented as mean ± SD (standard deviation) or mean ± SEM (standard error of the mean). Normality was assessed using the Shapiro-Wilk test; homogeneity of variance by Levene's test. For two-group comparisons, unpaired Student's *t*-test (normal, equal variance), Welch's *t*-test (normal, unequal variance), or Mann-Whitney U-test (non-normal) was applied. Multi-group comparisons employed one-way ANOVA with Tukey's or Bonferroni *post hoc* tests (parametric) or Kruskal-Wallis with Dunn's test (non-parametric). Longitudinal body weight trajectories were additionally evaluated using a linear mixed-effects model (group × time; random intercept for mouse) in R. Alpha diversity used Kruskal–Wallis; beta diversity significance used PERMANOVA (999 permutations). LEfSe identified differential taxa (LDA > 2, *p* < 0.05). Metabolomic multivariate modeling (PCA/OPLS-DA) was performed in MetaboAnalyst 5.0 (R backend; ropls package), and OPLS-DA robustness was assessed by permutation testing (*n* = 200). All *p* values were two-tailed; multiple testing in metabolomics, correlation networks, and cytokine panels was controlled by FDR unless specified. Statistical analyses were performed in GraphPad Prism v9.0 and R (version 4.3.2).

## 3 Results

### 3.1 Effect of COS on body weight

Over the 22-week experimental period, body weight trajectories diverged among the young control (YCK), elderly control (ECK), and COS-treated elderly (ECO) groups (n = 10 per group; no attrition). As shown in Figure 1, both YCK and ECK mice exhibited progressive weight gain (ECK: 29.03 ± 0.54 g to 34.37 ± 2.97 g; YCK: 25.76 ± 0.30 g to 35.57 ± 5.07 g). The noticeable weight gain observed in the YCK group between weeks 21 and 27 is attributed to the physiological maturation and natural adiposity accumulation characteristic of male C57BL/6 mice at this developmental stage. In contrast, ECO mice showed only a modest increase (28.97 ± 0.54 g to 30.61 ± 0.89 g), indicating relative weight stabilization. Terminal body weight in the ECO group was significantly lower than in both ECK and YCK (p < 0.05, one-way ANOVA), suggesting that COS attenuated age-associated weight gain. (Figure 1A, experimental design; Figure 1B, longitudinal body weights; Figure 1C, body-weight gain rate.) Furthermore, food intake was monitored throughout the experiment, and no significant differences were observed between the ECK and ECO groups (data not shown), ruling out appetite suppression as a cause for the observed weight difference.

### 3.2 Serum cytokines analysis

**Figure 2:**
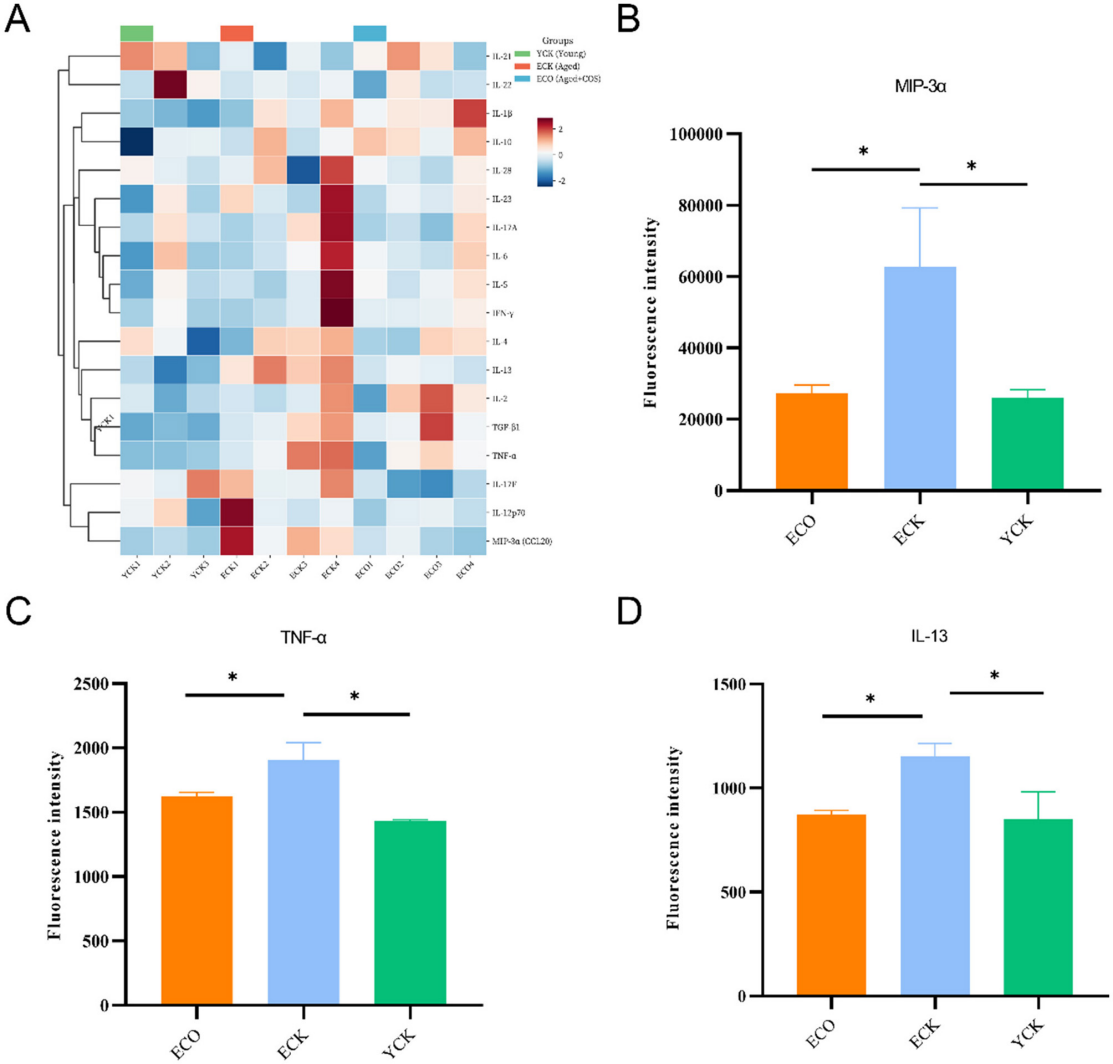
Differential cytokine expression in ECO, ECK, and YCK groups. **(A)** Heatmap illustrating clustered expression profiles of serum levels of all 18 cytokines, measured by the RayBio Quantibody Mouse Th17 Array 1 across samples. Data are presented as normalized fluorescence intensity. Serum levels of **(B)** MIP-3α (CCL20), **(C)** TNF-α, and **(D)** IL-13 in ECO, ECK, and YCK groups. Cytokine concentrations were quantified by ELISA using sera from mice (*n* = 3–4 per group. Bars represent mean ± SEM. One-way ANOVA followed by Fisher's uncorrected least significant difference (LSD) *post hoc* test was used to evaluate group differences. Statistical significance: ^*^p < 0.05. Asterisks indicate significance levels. ECO, ECK, and YCK denote: ECO, aged COS; ECK, aged control; YCK, young control.

To assess aging-associated systemic inflammation and the impact of COS, serum cytokines were profiled (*n* = 5–8; three independent experiments pooled). COS intervention significantly reduced MIP-3α, IL-13, and TNFα in ECO compared with ECK (*p* < 0.05), with levels approximating those in YCK (Figure 2). Anti-inflammatory or Th17-related cytokines such as IL-10 and IL-17 showed no significant differences. Data are presented as mean ± SEM and analyzed by one-way ANOVA followed by Fisher's LSD (uncorrected). These findings indicate that COS partially restores immune homeostasis in aged mice.

### 3.3 Effects of COS treatment in aged mice (p53/p21 pathway)

The p53–p21 axis orchestrates DNA-damage surveillance, stress-adaptive cell-cycle checkpoints, and senescence-associated programs. Given its central role in tissue aging, we examined whether COS modulates p53/p21 signaling in aged mice.

Immunohistochemical staining for p53 and p21 was performed on kidney and brain sections from YCK, ECK, and COS-treated elderly (ECO) mice. Nuclear staining intensity was semi-quantified using the H-score method (0-300). Data were analyzed by one-way ANOVA with *post hoc* Fisher's LSD.

**Figure 3:**
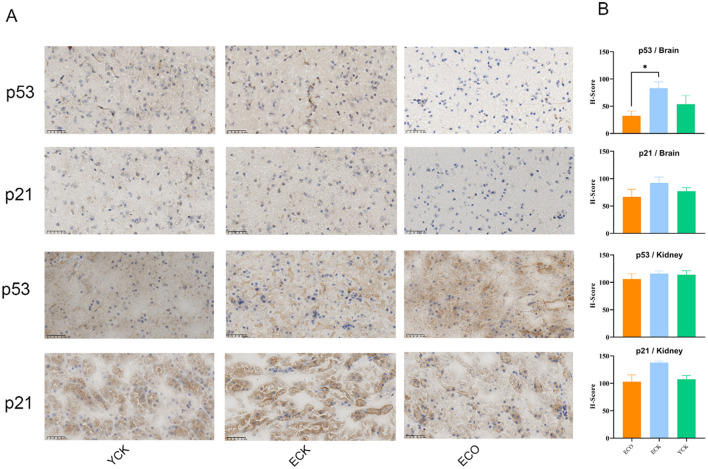
**(A)** Immunohistochemical expression of p53 and p21 in kidney and brain tissues of different treatment groups. Representative IHC micrographs showing p53 and p21 staining in the kidney and brain across the ECK, ECO and YCK (scale bar: 50 μm). **(B)** Quantification of p53 and p21 immunoreactivity using the *H*-score method (theoretical range 0–300). Data are presented as mean ± SEM (*n* = 5 per group). Statistical analysis was performed using *t-*tests, *p*-value < 0.05, ^*^p < 0.05.

Compared with elderly controls (ECK), COS treatment markedly down-regulated nuclear immunoreactivity of both p53 and its downstream effector p21 in kidney and brain tissues (Figure 3A). Semi-quantitative analysis demonstrated significant reductions (p53: *p* < 0.01; Figure 3B). YCK levels were comparable to ECO (*p* > 0.05 for both markers). No excessive background or aberrant cytoplasmic staining was observed, supporting specificity of the signal.

The coordinated down-regulation of p53 and p21 is consistent with a reduced activation burden of the stress-induced senescence axis and may reflect diminished chronic stress signaling rather than impairment of checkpoint control. Functional assays (e.g., DNA damage markers, cell-cycle profiling, SASP factor secretion) will be required to determine whether these molecular changes translate into improved tissue surveillance or regenerative capacity.

### 3.4 COS modulates fecal short-chain fatty acids (SCFAs)

**Figure 4:**
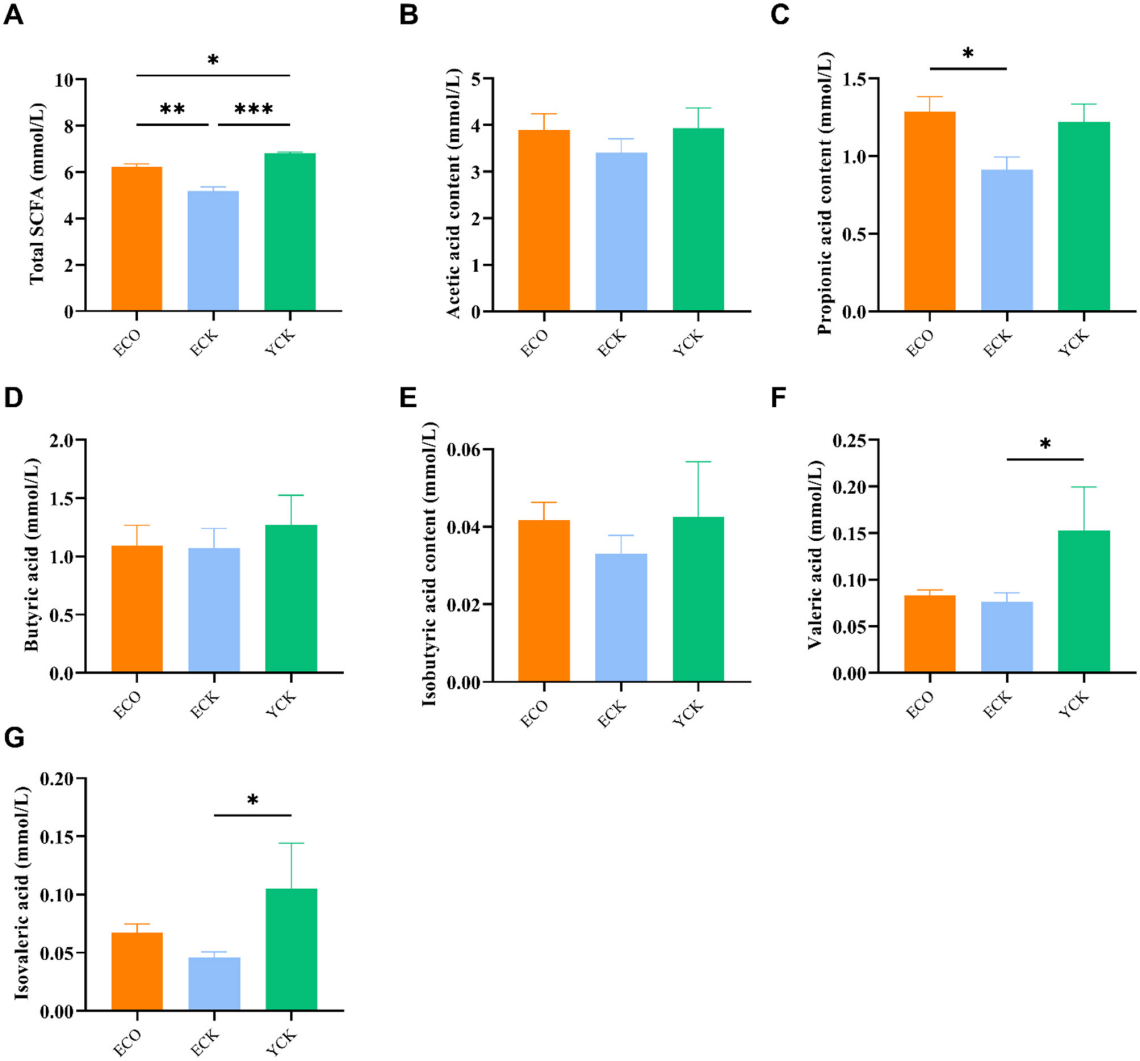
Short-chain fatty acid (SCFA) concentrations in cecal contents of young and aged mice with or without chitosan oligosaccharide (COS) intervention. **(A)** total SCFA, **(B)** Acetic acid, **(C)** Propionic acid, **(D)** Butyric acid, **(E)** Isobutyric acid, **(F)** Valeric acid, and **(G)** Isovaleric acid. SCFAs were quantified by GC-FID and expressed in mmol/L. Data are presented as mean ± SEM (YCK *n* = 10; ECK *n* = 10; ECO *n* = 15). Statistical analysis was performed using one-way ANOVA followed by Kruskal–Wallis with Dunn's correction after assessing normality and homogeneity of variance. ^*^p < 0.05, ^**^p < 0.01, ^***^p < 0.001. YCK, young control (3-month-old at baseline); ECK, aged control (10-month-old at baseline, 18-month-old at endpoint); ECO, aged + COS intervention.

Cecal SCFA profiles (Figure 4) demonstrated partial metabolic remodeling by COS. Acetate levels were similar between YCK and ECO and were only numerically higher than ECK (not statistically significant). Propionate was significantly elevated in both YCK and ECO relative to ECK (*p* < 0.05). Butyrate showed a non-significant trend toward higher levels in ECO. Iso-butyrate did not differ among groups. Valerate and isovalerate were significantly higher in YCK than in both ECK and ECO (*p* < 0.05). These data indicate that COS selectively modulates specific SCFAs—particularly propionate—rather than globally shifting the entire SCFA pool (one-way ANOVA).

### 3.5 Gut microbiota diversity and composition

**Figure 5:**
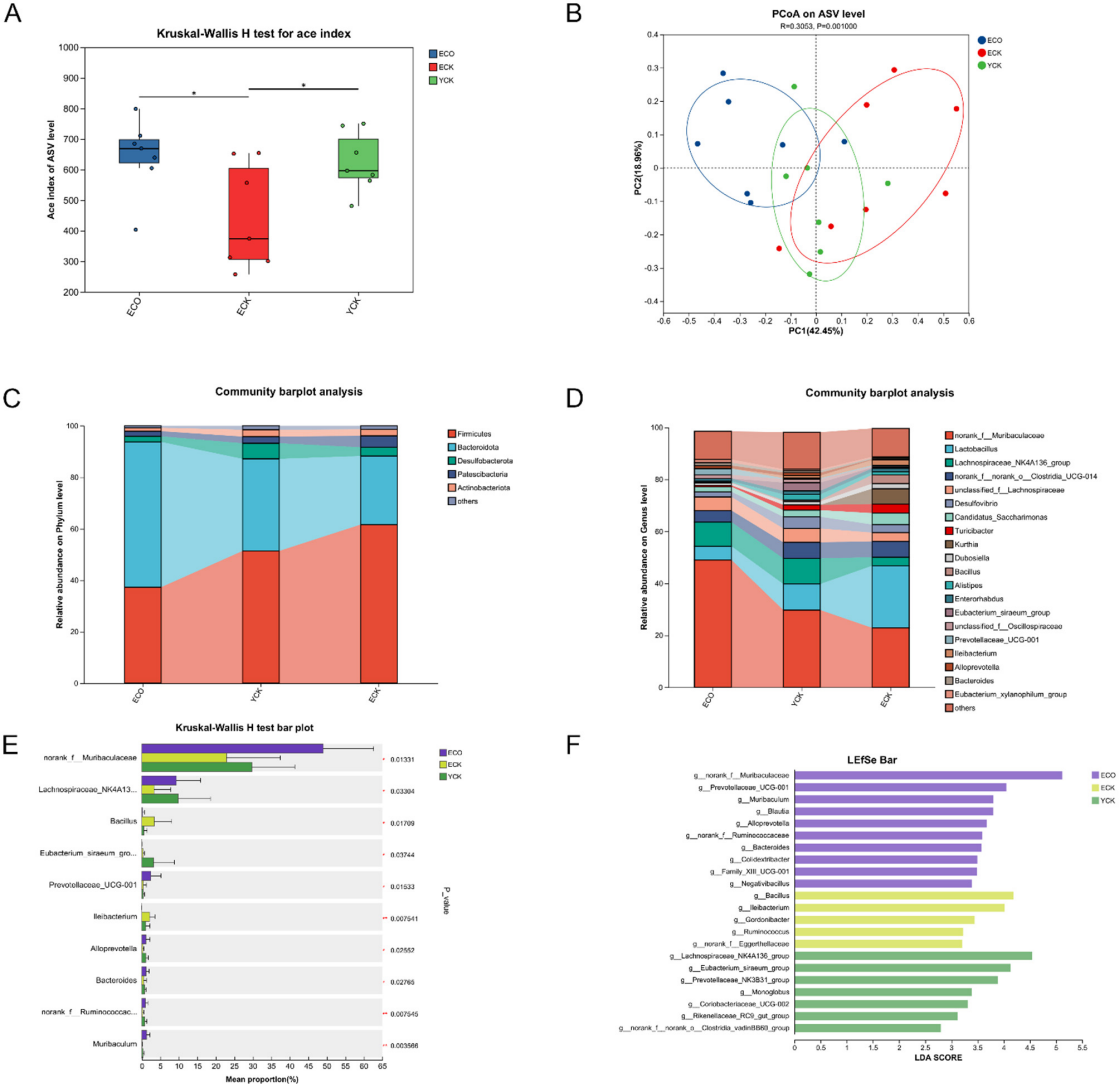
Chitosan oligosaccharide (COS) intervention modulated the gut microbiota structure in aged mice. **(A)** Alpha diversity indices (Sobs, Chao, Shannon, and Goods coverage) compared among groups (YCK *n* = 10; ECK *n* = 10; ECO *n* = 15) using the Kruskal–Wallis test with Dunn's *post hoc* correction. **(B)** Principal Coordinate Analysis (PCoA) based on Bray–Curtis distances (PERMANOVA, 999 permutations). **(C)** Relative abundance at the phylum level (stacked bar plot; low-abundance phyla merged as “Other”). **(D)** Relative abundance of the top 20 genera. **(E)** Genera showing significant differences among groups (Kruskal-Wallis *H* test with Dunn's *post hoc* test). **(F)** LEfSe analysis identifying differentially enriched taxa (LDA score > 2, *p* < 0.05). Cecal microbiota were profiled by 16S rRNA gene sequencing (V3–V4 region; Illumina MiSeq, paired-end 2 × 250 bp). ^*^p < 0.05, ^**^p < 0.01. YCK, young control; ECK, aged control; ECO, aged + COS.

Gut microbiota diversity and composition were altered by aging and partially modulated by COS intervention (Figure 5). Alpha-diversity analysis showed that richness (ACE index; Figure 5A) declined with age: both elderly control (ECK) and COS-treated elderly (ECO) mice displayed significantly lower ACE values than young controls (YCK) (*p* < 0.05, Kruskal–Wallis), while ECO exhibited a non-significant trend toward partial recovery vs. ECK. In contrast, Shannon and Simpson indices (Figures 5B, C) did not differ among groups, indicating that evenness was largely preserved despite age-related loss of low-abundance taxa.

Beta-diversity (PCoA; Figure 5D) revealed clear separation among YCK, ECK, and ECO, supporting the conclusion that aging reshaped overall community structure and that COS further shifted the aged microbiota rather than simply reverting it to a youthful baseline.

At the phylum level (Figure 5E), aging (ECK vs. YCK) was associated with increased *Firmicutes, Actinobacteriota*, and *Patescibacteria*, together with a reduction in *Bacteroidota*. COS intervention partially countered these changes: ECO animals showed decreased relative abundances of the age-elevated *Firmicutes, Actinobacteriota*, and *Patescibacteri*a, while *Bacteroidota* increased from 15.29% (ECK) to 31.22% (ECO), suggesting attenuation of age-associated compositional drift.

Genus-level profiling (Figure 5F) highlighted taxa consistent with functional remodeling. Aging reduced norank_f_*Muribaculaceae* (14.28% vs. 35.54% in YCK) and *Lachnospiraceae*_NK4A136 (3.61% vs. 4.42%), while markedly increasing *Lactobacillus* (28.91% vs. 10.66%). COS treatment further elevated norank_f_*Muribaculaceae* to 52.83% and *Lachnospiraceae*_NK4A13 to 7.16%, and sharply decreased *Lactobacillus* to 2.31%. These directional shifts collectively indicate a microbial architecture trending toward a “younger” or metabolically favorable ecological signature enriched in putative fiber-degrading and SCFA-associated lineages.

LEfSe analysis (Figures 5G, H; LDA threshold = 2) identified *Lactobacillus* (LDA = 5.14) as a biomarker enriched in ECK, norank_f_*Muribaculaceae* (LDA = 5.30) and *Prevotellaceae*_UCG-001 (LDA = 4.13) as enriched in ECO, and *Lachnospiraceae*_NK4A136 (LDA = 4.62) as enriched in YCK. These discriminant taxa plausibly interface with SCFA production and host metabolic signaling, providing a mechanistic bridge between compositional remodeling and downstream immune-metabolic effects.

### 3.6 COS modulates senescence-associated metabolic pathways

**Figure 6:**
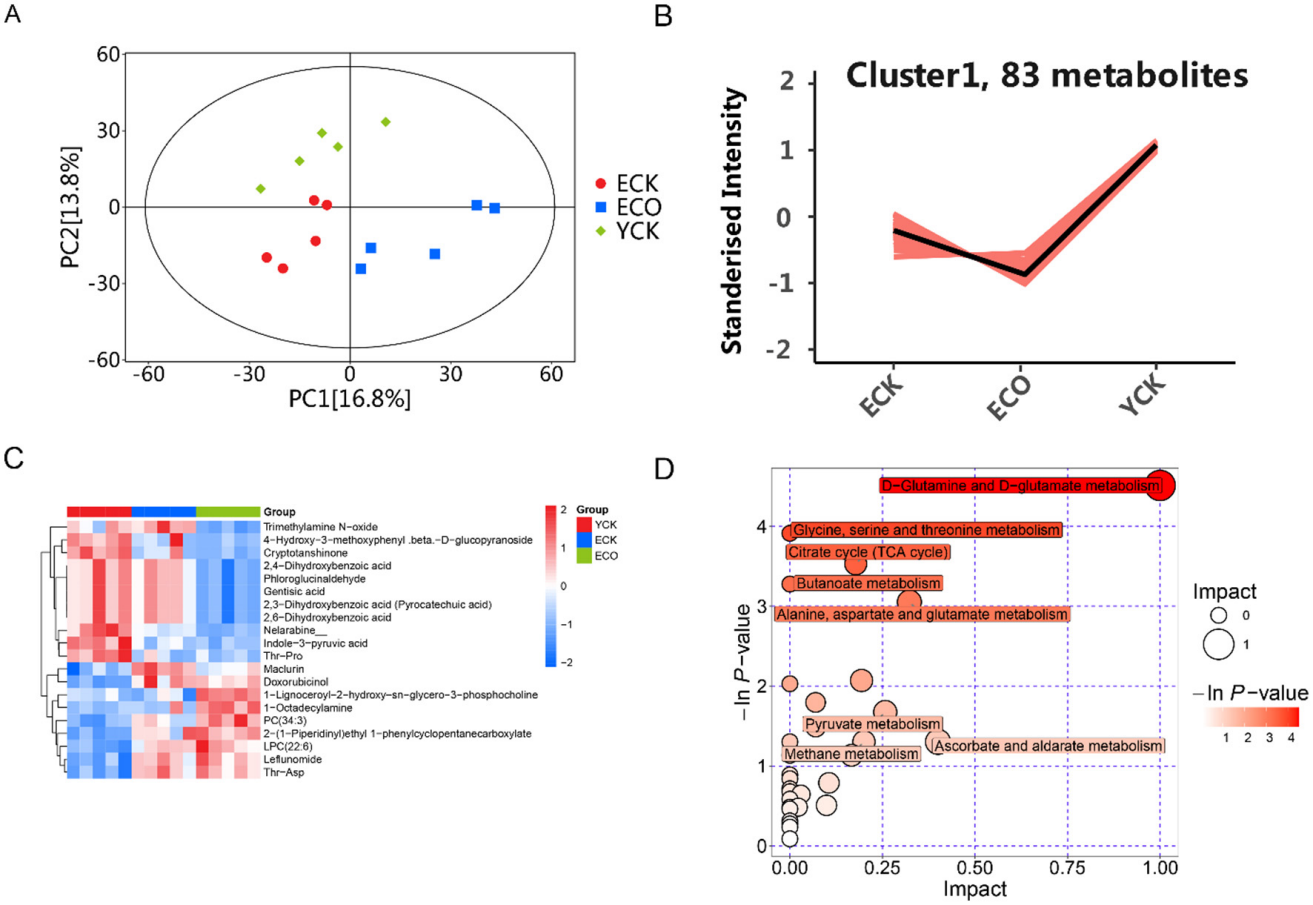
Chitosan oligosaccharide (COS) intervention modulated the serum metabolomic profile in aged mice. **(A)** PCA score plot showing global metabolic clustering (YCK *n* = 10; ECK *n* = 10; ECO *n* = 15). **(B)** K-means clustering plot. **(C)** Hierarchical clustering heat map of differential metabolites after Z-score normalization (rows: metabolites; columns: samples). **(D)** KEGG pathway enrichment and topology analysis bubble plot: the x-axis shows pathway impact, bubble size is proportional to the impact value, and the color scale represents the negative natural logarithm of the *p*-value. Only representative significant pathways (*p* < 0.05 or FDR < 0.05) are shown. Serum metabolites were profiled by untargeted LC-MS. YCK, young control; ECK, aged control; ECO, aged + COS; COS, chitosan oligosaccharide.

Untargeted metabolomic profiling revealed that COS modulated age-associated metabolic remodeling in elderly mice (Figure 6). Multivariate ordination showed clear separation among elderly control (ECK) \ COS-treated elderly (ECO) groups and young control group (YCK) in the PCA score plot (Figure 6A), K-means clustering (*k* = 9) of standardized metabolite intensities across the ECK, ECO, and YCK groups identified distinct expression patterns, with cluster sizes ranging from 11 to 83 metabolites (Supplementary Figure S1). Notably, Clusters 1 (83 metabolites), 3 (57 metabolites), and 9 (22 metabolites) exhibited aging-induced upregulation in ECK relative to YCK, which was significantly reversed by COS treatment in EI, restoring levels toward youthful states (ANOVA, *p* < 0.01 for inter-group differences). In contrast, Clusters 2, 6, and 7 showed partial amelioration, while Clusters 4, 5, and 8 displayed neutral or divergent responses, suggesting pathway-specific effects. These patterns align with COS modulation of oxidative stress and energy metabolism pathways, as detailed in subsequent enrichment analyses (see Supplementary Figure S1 for full cluster profiles). K-means clustering reveals Cluster 1 (*n* = 83 metabolites) as a key responder to COS intervention, reversing aging-induced upregulation (Figure 6B).

Hierarchical clustering of standardized intensities for 20 differentially abundant metabolites revealed clear group separation among ECK, ECO, and YCK samples (Figure 6C). Metabolites clustered into two major groups: the upper cluster (e.g., Trimethylamine N-oxide [TMAO], Cryptotanshinone, and phenolic acids like 2,4-Dihydroxybenzoic acid) showed upregulation in ECK (positive intensities) and downregulation in ECO and YCK (negative intensities), consistent with aging-induced oxidative stress and inflammation. In contrast, the lower cluster [e.g., Indole-3-pyruvic acid, Thr-Pro dipeptide, and lysophosphatidylcholines [LPCs] like LPC (22:6)] exhibited downregulation in ECK and restoration toward YCK levels in ECO, aligning with K-means patterns in Clusters 1, 3, and 6 (Supplementary Figure S1). These shifts underscore COS's modulation of gut microbiota-derived metabolites, potentially alleviating cognitive decline and neuroinflammation via enhanced antioxidant pathways and blood-brain barrier permeability. Pathway enrichment confirmed involvement in amino acid metabolism (e.g., glutamine/glutamate) and oxidative stress responses (Figure 6E), supporting COS as a therapeutic agent for metabolic reprogramming in aging.

### 3.7 Summary of findings

In aged mice, COS supplementation modestly dampened late-phase body weight gain and selectively suppressed circulating pro-inflammatory mediators (MIP-3α, IL-13, and TNFα), indicating a systemic anti-inflammatory shift. Concomitantly, COS attenuated the renal and cerebral p53–p21 senescence axis, suggesting mitigation of cellular stress signaling in organs vulnerable to age-associated functional decline. These immune–senescence adjustments occurred alongside partial reshaping of fecal short-chain fatty acid profiles, most notably an elevation in propionate compared with untreated aged controls.

At the ecological level, COS reconfigured gut microbial richness and community structure, increasing the relative abundance of norank_f_*Muribaculaceae* toward or beyond the young level and counteracting age-linked shifts across multiple phyla and genera. This microbial re-calibration paralleled broad metabolomic reprogramming encompassing amino acid, nitrogen, and carbon metabolism, with glutamine/glutamate-centered routes emerging as potential metabolic hubs. Together, the coordinated modulation of host inflammatory tone, senescence pathways, microbial ecology, and metabolite net-works supports an integrative model in which COS confers anti-aging benefits through coupled immune-metabolic and microbe–host regulatory mechanisms.

## 4 Discussion

This integrated study demonstrates that COS mitigates aging-related gut dysbiosis, metabolic dysregulation, low-grade inflammation, and cellular stress signaling in naturally aged mice. COS increased *Bacteroidota* and norank_f_*Muribaculaceae* abundance to-ward or beyond young levels—taxa associated with SCFA production, mucosal energy supply, and barrier integrity (11)—while reducing Firmicutes and Lactobacillus enrichment observed in untreated aged animals, suggesting partial reversal of age-associated compositional drift (12).

Untargeted metabolomics revealed multiple discriminant metabolites, including tri-methylamine N-oxide (TMAO)—a compound previously implicated in cardiometabolic and vascular aging phenotypes (13)—and several putatively annotated aromatic and nitrogenous small molecules (e.g., phenolacine, phloroglucinaldehyde, melabarine). The enrichment of pathways included carbon metabolism, taurine and hypotaurine metabolism, D-glutamine and D-glutamate metabolism, as well as pathways for aromatic amino acids like phenylalanine, tyrosine, and tryptophan, alongside broader amino acid, nitrogen, and lipid metabolic remodeling, aligning with a microbiota-metabolite interaction framework. Consistent with these shifts, COS modestly attenuated body weight gain, congruent with prior anti-obesity or weight-stabilizing properties (15). It is important to note that COS treatment did not cause weight loss below baseline but rather prevented the excessive age-associated adiposity observed in the control group. In C57BL/6 mice, this attenuation of weight gain is associated with improved insulin sensitivity and reduced inflammation, consistent with a health-promoting effect. Notably, COS attenuated renal and cerebral p53-p21 axis immunoreactivity (down-regulation of both p53 and p21), consistent with a reduced chronic activation burden of canonical DNA damage/stress-response checkpoints that often reinforce inflammation and metabolic rigidity in advanced age (3).

Immunologically, modulation of circulating cytokines included selective suppression of TNF-α, IL-13, and MIP-3α (CCL20). Although CCL20 elevation has been linked to chronic inflammatory milieus and chemokine-driven leukocyte recruitment (16), its nuanced regulation may reflect context-dependent immune set-point recalibration. Mechanistically, CCL20/CCR6 signaling is known to potentiate pro-inflammatory cascades in rheumatoid arthritis via CCR6+ monocyte recruitment and IL-6 induction (2, 19), and can be transcriptionally amplified by IL-1β, TNF-α, and IL-17 through EGFR-Ras axis engagement with links to cellular senescence programming (21). Its involvement in upper airway inflammatory remodeling further underscores its pleiotropy (22, 23). The observed attenuation of TNF-α alongside modulation of IL-13 fits within a complex cytokine net-work influencing fibrosis, matrix turnover, and metabolic inflammation (24, 25), while ex-ternal bioactives exemplify how suppression of TNF-α/IFN-γ pathways can alleviate age-related dermal inflammatory damage (26). Within this landscape, the down-regulation of p53-p21 may synergize with lowered pro-inflammatory tone to reduce accumulation of senescence-associated secretory phenotypes (SASP) and preserve metabolic flexibility.

Microbiome-metabolome coupling emerged as a central axis. In this study, we analyzed cecal contents to characterize the direct alterations in the gut ecosystem (microbiota and SCFAs) and serum to capture the systemic metabolic signatures, thereby reconstructing the functional trajectory of the Microbiota-Metabolite-Immune axis from the gut to the circulation. Aging is typified by erosion of SCFA-producing taxa and diminished luminal SCFA pools (4, 27). COS-mediated increase of norank_f_*Muribaculaceae* coincided with increased fecal propionate, a metabolite with expanding relevance to systemic energy partitioning, intestinal barrier function, and host metabolic resilience.

Propionate can serve as a peripheral energy substrate and modulate gluconeogenic and lipid pathways after trans-epithelial absorption (28), and *Muribaculaceae*-derived saccharolytic fermentation has been linked to host longevity trajectories via SCFA provisioning (29). Emerging evidence associates propionate flux with regulation of NAD+ metabolic circuitry, potentially influencing sirtuin activity and redox homeostasis (30); elevated propionate and fiber-associated metabolic remodeling in certain longevity cohorts further implicate SCFA ecology in cognitive preservation, antioxidant capacity, and restrained mucosal inflammation (31). Potential FFAR2/FFAR3 and HDAC-inhibitory signaling could underlie part of the observed immunometabolic recalibration (32), while integration with NF-κB and inflammasome modulation pathways remains to be explicitly resolved in this model.

Comparison with the D-galactose accelerated aging paradigm underscores model-dependent translational value. The D-galactose regimen induces rapid oxidative and glycoxidative stress impacting mitochondrial proteostasis and redox balance (14), facilitating high-throughput nutraceutical screening but narrowing mechanistic breadth via a dominant ROS/AGE axis (33). By contrast, the natural aging model employed here recapitulates multifactorial low-grade inflammation, progressive microbial ecological drift, and composite metabolic remodeling, thereby offering greater relevance for late-life hu-man physiology and chronic immunometabolic transitions pertinent to COS translation.

The convergence of microbiota restructuring, SCFA pattern recalibration, cytokine suppression, and attenuation of the p53-p21 senescence program differentiates COS's effects in a chronic aging milieu from hypothetical, potentially more acute or restricted responses anticipated in D-galactose models. Future head-to-head comparative studies incorporating synchronized sampling, equivalent dosing regimens, and standardized multi-omic pipelines would clarify conserved vs. model-specific intervention nodes.

Strengths of this study include integration of microbiomics and metabolomics to link ecological restructuring with systemic biochemical outputs, and parallel assessment of immunosenescence and canonical stress-axis markers. Nonetheless, several limitations constrain mechanistic granularity: (i) incomplete SCFA quantitation (e.g., absence of absolute concentrations and full acetate: propionate: butyrate stoichiometry), (ii) lack of targeted validation (MS/MS reference standards) for all discriminatory metabolites (some remain putative, MSI Level 3), (iii) omission of detailed pathway effect sizes and directionality for specific metabolic nodes, (iv) absence of functional assays probing SCFA-GPCR (FFAR2/FFAR3), GPR109A, or NF-κB signaling cascades, and (v) moderate sample size without longitudinal multi-timepoint profiling. Additionally, while this study focused on the endpoint effects to correlate with tissue pathology, future studies incorporating longitudinal fecal sampling are needed to dynamically track the temporal shifts in microbial guilds during the aging process. Finally, this study exclusively used male mice to minimize the confounding effects of the estrous cycle on microbiome and metabolic variability; however, this limits the generalizability of the findings, and future studies must include female cohorts to evaluate potential sex-dimorphic responses to COS intervention.

Future work should incorporate targeted SCFA panels, stable isotope tracing to map nitrogen and carbon flux through glutamine/glutamate and taurine circuits, single-cell or spatial transcriptomics to localize senescence attenuation, and intervention arms including antibiotic depletion or gnotobiotic colonization to assign causality to microbial communities. Parallel evaluation of mitochondrial bioenergetics, NAD+ salvage enzyme activity, and chromatin accessibility could further elucidate links between attenuated p53–p21 activity and immunometabolic plasticity. Expanded cohorts and extended intervention durations will also be critical to assess durability and dose-response relationships, and to bench-mark efficacy against established geroprotective agents (e.g., caloric restriction mimetics) (34).

In summary, COS exerts multi-layered anti-aging effects characterized by (i) increases in SCFA-associated microbial taxa, (ii) propionate-associated metabolic remodeling across carbon, nitrogen, and amino acid pathways, (iii) selective suppression of inflammatory chemokines and cytokines, and (iv) attenuation of renal and cerebral p53-p21 senescence signaling. These convergent shifts support an integrative immunometabolic and microbiota–host modulatory mechanism and position COS as a promising candidate for further translational geroscience evaluation.

## 5 Conclusions

Chitosan oligosaccharide (COS) supplementation in naturally aged mice produced a multi-layered geroprotective profile characterized by: (i) increased SCFA-associated taxa (e.g., *Bacteroidota* and norank_f_*Muribaculaceae*) toward or beyond young levels with concurrent rebalancing of age-shifted Firmicutes; (ii) remodeling of metabolic signatures, including modulation of amino acid, nitrogen, and lipid pathways and a propionate-associated microbial fermentation pattern; (iii) immunoregulatory adjustment reflected by suppression of MIP-3α (CCL20) and attenuation of pro-inflammatory cytokines (e.g., TNF-α, IL-13); (iv) attenuation of the p53–p21 senescence axis in target tissues; and (v) attenuation of overall body weight gain. Integrated microbiome-metabolome analysis supports a coordinated microbiota–metabolite–immune interaction through which COS may promote SCFA-linked metabolic flexibility and dampen inflammation.

Current data gaps-absence of full quantitative SCFA panels, targeted metabolite validation, receptor (FFAR2/FFAR3, GPR109A) and NF-κB signaling assays, and longitudinal multi time-point resolution—limit definitive pathway attribution. Addressing these limitations, alongside head-to-head evaluation in both natural and accelerated aging models and dose-response optimization, will be essential to consolidate causality and translational relevance. Overall, the present findings position COS as a microbiota- and metabolism-modulating candidate for future healthy aging interventions and potential combinatorial geroscience strategies.

## Data Availability

The data presented in this study are publicly available. The data can be found here: https://www.ncbi.nlm.nih.gov, accession PRJNA1328410.
