# Supplementary material for: Impact of chitosan oligosaccharide on microbiota-metabolite-immune axis in natural aging
Source: Front Nutr. 2026 Jan 23;12:1722269. doi: 10.3389/fnut.2025.1722269 (PMC12876160; doi:10.3389/fnut.2025.1722269)
Supplement: Supplementary file 1 [file Presentation_1.pdf]

Supplementary Material

1 Supplementary Figures and Tables

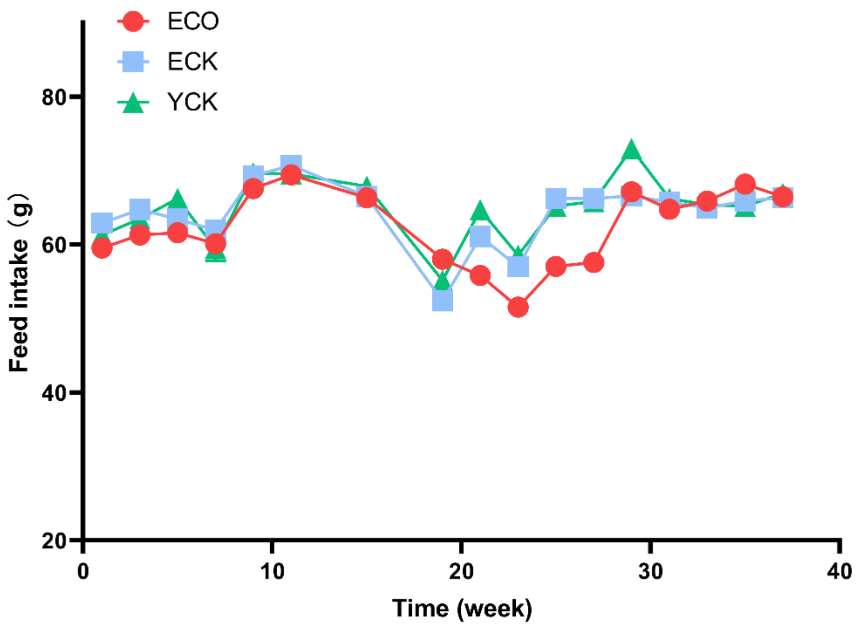

Supplementary Figure 1. Results of feed intake during day and night across ECO, ECK and YCK groups.

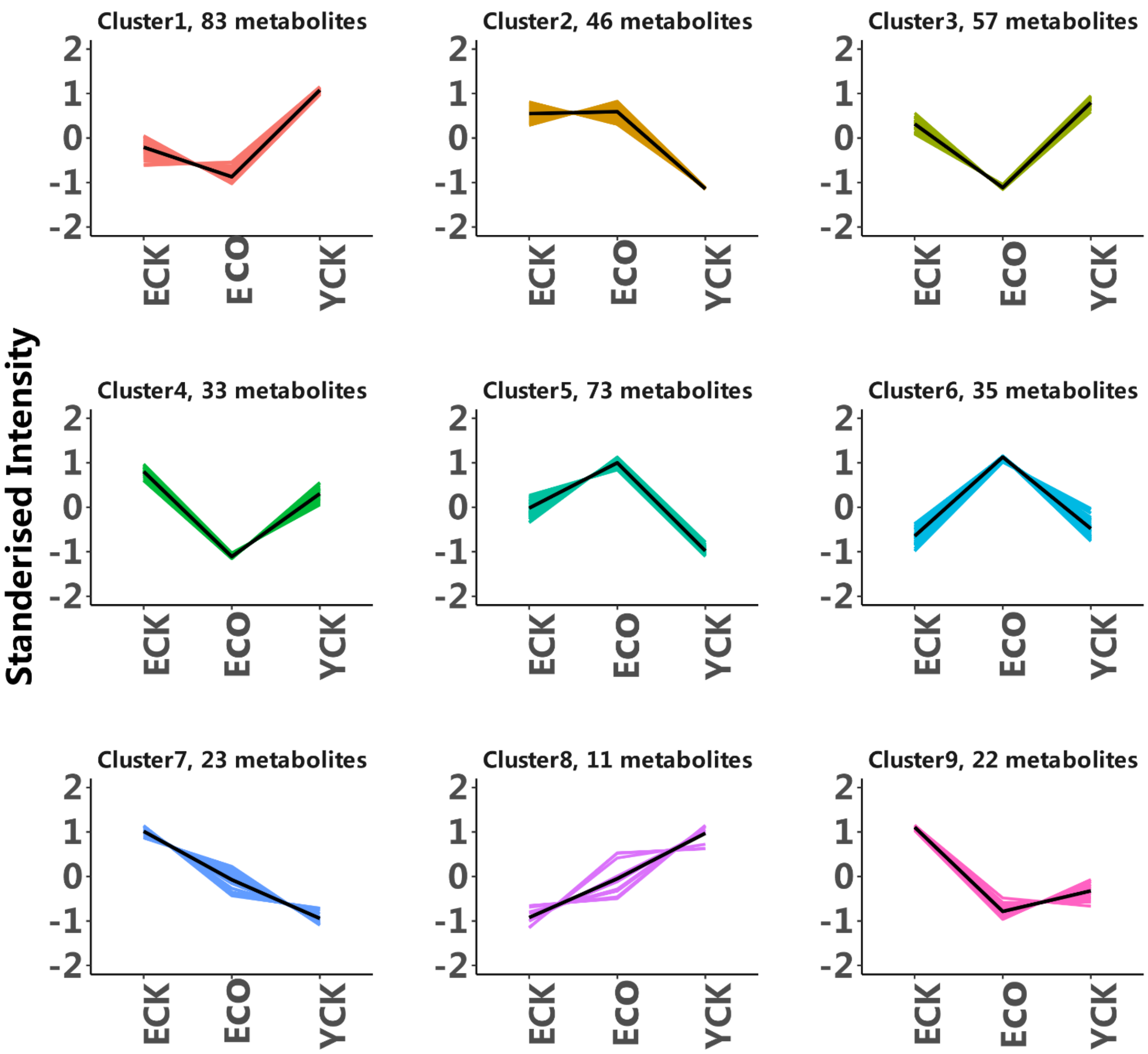

Supplementary Figure 2. K-means clustering of metabolite intensity profiles across ECK, ECO, and YCK groups. K-means clustering (k=9) of standardized metabolite intensities from non-targeted metabolomics in mouse models, grouped by ECK, ECO, and YCK. Each panel represents one cluster with the number of metabolites indicated (e.g., Cluster 1: 83 metabolites). Line plots show mean standardized intensity (± SEM, if applicable) on the y-axis (-2 to 2) for each group on the x-axis. Clusters demonstrate distinct patterns: e.g., Cluster 1 and 3 exhibit aging-induced upregulation (higher in ECK) reversed toward youthful levels (YCK) by COS (ECO); Cluster 6 shows partial restoration of downregulation.
